# Identification of flux trade-offs in metabolic networks

**DOI:** 10.1038/s41598-021-03224-9

**Published:** 2021-12-10

**Authors:** Seirana Hashemi, Zahra Razaghi-Moghadam, Zoran Nikoloski

**Affiliations:** 1 Bioinformatics, Institute of Biochemistry and Biology, University of Potsdam, 14469 Potsdam, Germany; 2 Systems Biology and Mathematical Modelling, Max Planck Institute of Molecular Plant Physiology, 14476 Potsdam, Germany

**Keywords:** Computational biology and bioinformatics, Systems biology

## Abstract

Trade-offs are inherent to biochemical networks governing diverse cellular functions, from gene expression to metabolism. Yet, trade-offs between fluxes of biochemical reactions in a metabolic network have not been formally studied. Here, we introduce the concept of absolute flux trade-offs and devise a constraint-based approach, termed FluTO, to identify and enumerate flux trade-offs in a given genome-scale metabolic network. By employing the metabolic networks of *Escherichia coli* and *Saccharomyces cerevisiae*, we demonstrate that the flux trade-offs are specific to carbon sources provided but that reactions involved in the cofactor and prosthetic group biosynthesis are present in trade-offs across all carbon sources supporting growth. We also show that absolute flux trade-offs depend on the biomass reaction used to model the growth of *Arabidopsis thaliana* under different carbon and nitrogen conditions. The identified flux trade-offs reflect the tight coupling between nitrogen, carbon, and sulphur metabolisms in leaves of C_3_ plants. Altogether, FluTO provides the means to explore the space of alternative metabolic routes reflecting the constraints imposed by inherent flux trade-offs in large-scale metabolic networks.

## Introduction

The expression of many traits in biological systems cannot be increased at the cost of decreasing others, pointing at trade-offs^1^. For example, the trade-off between the rate and yield of adenosine triphosphate (ATP) production has been used to explain why evolution may work to select a less efficient pathway when cells compete for a shared resource^2^. This question is an instance of the more general trade-off between enzyme efficiency and substrate efficiency in metabolism at the core of the trade-off between growth rate and yield observed in microbes^3^. The growth rate-yield trade-off has been studied by using different modelling approaches, including: (i) elementary flux modes, combined with enzyme-cost estimations^3^, (ii) fine-grained genome-scale models of metabolism and gene expression^4^, and (iii) constraint-allocation flux balance analysis^5^, combining mass balance and proteomic constraints. In addition, by using fluxes from multiple experiments in *E. coli* and constraint-based modelling, it has been shown that flux states arise due to the trade-off between optimality under a given condition and minimal adjustments between conditions^6^. Indeed, a recent study has uncovered a universal trade-off between growth rate and adaptability in *E. coli*, *Bacillus subtilis*, and *Saccharomyces cerevisiae*^7^. These examples indicate that trade-offs between multiple traits often arise due to the optimization of multiple objectives; these traits may be recovered by considering the extreme points of a convex hull in a principal component space containing observed phenotypes^1^.

A simple way to explain trade-offs between traits is the resource acquisition-allocation model^8,9^, referred to as the Y-model^10^. The Y-model postulates that two traits, $X_{1}$ and $X_{2}$ taking non-negative values, are shaped by the allocation of resources from a common pool, $T$, determined by the acquisition. As a result, it holds that $X_{1} + X_{2} = T$ which implies that at a fixed acquisition (i.e., $T$ held constant), the allocation of some proportion of the acquired resource to $X_{1}$, restricts the value of $X_{2}$ and leads to a negative correlation between the traits in trade-offs. In contrast, if the acquisition, $T$, is allowed to vary, the Y-model explains that two traits can be in a trade-off but may also show positive correlations^10^. The Y-model indicates that the traits can be considered to be in trade-off if: (i) they show some level of phenotypic plasticity, i.e., different expressions under different environments, and (ii) if there exists a non-negative linear combination that corresponds to a resource. We refer to a trade-off as *absolute* if the resource is robust, in the sense that it exhibits no phenotypic plasticity over a range of environments (i.e., $T$ is held constant); otherwise, we refer to the trade-off as *relative*.

Fluxes in a metabolic network can be readily studied in the framework provided by the Y-model. The flux of a reaction is determined by the interplay between metabolites acting as substrates and/or regulators of enzymes that catalyse the reaction. Since metabolites participate in multiple reactions and enzymes can catalyse several reactions^11^, the fluxes in a metabolic network may be shaped by trade-offs that arise due to competition for these molecular resources. One way to determine fluxes in trade-off is to use flux sampling by imposing realistic constraints on growth and nutrient uptake^12,13^. Negative correlations can then reveal trade-offs between pairs of fluxes; however, this approach cannot be easily expanded to larger subsets of fluxes. Furthermore, there is no constraint-based approach that utilizes constraints from a given metabolic network structure with additional constraints on nutrient uptake to determine trade-offs between metabolic fluxes.

Here, we devise a two-step constraint-based approach, termed FluTO, that identifies and enumerates all *absolute* trade-offs between reaction fluxes, given certain constraints on input fluxes. We apply the approach in different scenarios with the large-scale networks of *E. coli*, *S. cerevisiae*, and *Arabidopsis thaliana* and demonstrate that absolute trade-offs between reaction fluxes are condition-specific. Our results indicate that there are inherent properties of the metabolic network structure that, together with realistic constraints, lead to the appearance of absolute flux trade-offs that limit flux routing through alternative metabolic pathways.

## Methods

### Metabolic network models and constraints

We apply our approach to stoichiometric metabolic models of three model organisms: the bacterium *E. coli*, the unicellular eukaryote *S. cerevisiae* and the model plant *A. thaliana*. We analysed the genome-scale metabolic model iJO1366 of *E. coli* str. K-12 substr. MG1655. This network consists of 1805 metabolites, 2583 reactions, and 1366 genes^14^. The model has the “core” and “wild-type” biomass reactions. The “wild-type” biomass reaction contains the precursors to all the typical wild-type cellular components of *E. coli*. In contrast, the “core” biomass reaction contains the precursors only to essential components^14^. We optimize the “wild type” biomass reaction and remove the “core” reaction from the model. We analysed 29 different growth conditions (11 different active carbon sources with a fixed growth rate)^15^. Applying flux variability analysis (FVA)^16,17^ with the model under the constraints from the aforementioned growth conditions resulted in fixed, fixed-sign variable, and sign-variable reactions (Supplementary Table 1). FVA did not result in fixed reactions for three growth conditions, so we determined trade-offs for 26 different conditions.

In a second case study, we used the same metabolic network model of *E. coli* with the wild type biomass reaction. We assigned a fixed value of -10 mmol/gDW/h for the active carbon source (one of 61 exchange carbon sources^18^, see Supplementary Table 2). We also set a fixed value of -0.9476 mmol/gDW/h for Phosphate exchange reaction, 3.15 mmol/gDW/h for ATP maintenance requirement reaction, 0.0931 mmol/gDW/h for ATP phosphoribosyltransferase reaction and 12.612 mmol/gDW/h for ATP synthase (four protons for one ATP) (periplasm) reaction. These values are obtained by rounds of FBA and FVA, as described in the results. Supplementary Table 2 shows the flux of reactions and reaction types.

We also investigated the genome-scale metabolic network, yeastGEM_v8.3.3, of *S. cerevisiae* with 2691 metabolites and 3963 reactions^19^. Like in the case of *E. coli*, above, we activated one carbon source among the 14 carbon sources and fixed its value to − 10 mmol/gDW/h (see Supplementary Table 3). We set the value of ATP synthase reaction to 39 mmol/gDW/h and the four O2 transport reactions to 8 mmol/gDW/h, 11.2 mmol/gDW/h, 19.9 mmol/gDW/h, and 0.9 mmol/gDW/h (see Supplementary Table 4 for reaction ratios and their types).

We used the AraCore model as representative metabolic network of *A. thaliana*, with 407 metabolites and 549 reactions^20^. The model has three different active biomass reactions: ‘Carbon limiting biomass’, ‘Nitrogen limiting biomass’, and ‘Light limiting biomass’. At three steps, we activated one of these three reactions and blocked the two others. The constraints that we added to the model include: allowing the ratio of sucrose to starch to be between 2.5 and 4 and the carboxylation/oxygenation ratio is between 1.5 and 4^21^. Under limiting carbon condition, we fixed the flux of ‘ATP’ reaction to 104.7678 mmol/gDW/h, and ‘Export O2’ to 88.2809 mmol/gDW/h; under nitrogen limiting conditions, we fixed the ‘ATP’ flux to 107.1427 mmol/gDW/h and ‘Export O2’ fixed to 87.5137 mmol/gDW/h. Finally, under light limiting conditions, the ATP' flux was set to 107.1427 mmol/gDW/h and the 'Export O2' to 84.1163 mmol/gDW/h (see Supplementary Table 5).

### Implementation

The proposed approach, FluTO, is implemented in MATLAB and is fully available at https://github.com/seirana/FluTo.

## Results and discussion

### Formulation and illustration of FluTO

**Figure 1:**
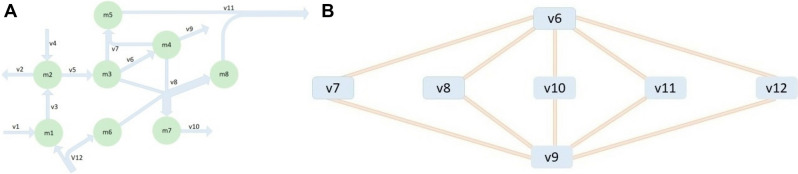
Illustration of flux trade-offs on a paradigmatic network. The toy network is composed of eight metabolites (green nodes) and 12 reactions (blue hyperedges) shown on panel (**A**). Under the constraints that reaction 5 has a flux fixed to 5 (a.u.) and the lower and upper boundaries for the fluxes of the remaining reactions are 0 and 10 (a.u.), there are ten trade-offs (orange edges), each involving two reactions (blue nodes), shown on panel (**B**).

We study a metabolic network, represented by a stoichiometric matrix, $N$, with $m$ metabolites and $r$ reactions, corresponding to the rows and columns of $N$. For instance, the network in Fig. 1A consists of eight metabolites and twelve reactions, of which reactions $R_{3}$, $R_{5}$, $R_{6}$, $R_{7}$, and $R_{8}$ are internal, and the remaining are exchange reactions. We assume that the network is at a steady-state, whereby $Nv = 0$. In addition, we impose fixed values for selected fluxes based on measured growth, nutrient uptake, and/or biochemically reasonable assumptions (see Methods). For instance, for the network in Fig. 1A, the lower and upper bounds of all reactions except for $R_{5}$ are set to 0 and 10 (arbitrary units), respectively, while reaction $R_{5}$ has a fixed flux of value 5 (a.u.). Together with the set of irreversible reactions, this results in a set of feasible steady-state fluxes $F = \left\{ {v\left| {Nv = 0 \wedge v_{{\min }} \le v \le v_{{\max }} } \right.} \right\}$ in which we aim to identify fluxes in an absolute trade-off. To this end, we devise a two-step approach, termed FluTO, that includes: (1) categorizing of metabolic reactions as blocked, fixed, and variable, and (2) finding a linear combination of rows that satisfy the constraints of the Y-model, namely that there exists a non-negative linear combination of reactions with variable fluxes which amounts to a constant.

To categorize the reactions based on the variability in the set of feasible steady-state flux distributions, $F$, we rely on FVA. A reaction is considered blocked if it does not carry flux in any flux distribution in $F$. A reaction is considered *fixed* if its flux takes a non-zero value with the same overall flux distributions in $F$. A reaction is considered a *fixed-sign variable* over the distributions in $F$ if it takes multiple values which are either non-negative or non-positive. We can consider that all fixed-sign variable reactions carry non-negative fluxes since, in the opposite case, the signs of the entries in the corresponding reaction vector in $N$ can be flipped. We refer to a reaction that is not blocked, fixed, or fixed-sign variable as a *sign-variable*. For instance, in Fig. 1A, reaction $R_{5}$ is fixed (to a value of 5), and all remaining reactions are fixed-sign variable (see Table 1). A sign-variable reaction is, by definition, reversible; it can be split into two irreversible reactions which can take any value in the applied lower and upper flux boundaries. As a result, splitting of sign-variable reactions into irreversible reactions has no effect of the identified absolute flux trade-offs, and their exclusion in FluTO is justified.

**Table 1:** The result of FVA on the reactions in the metabolic model of Fig. 1A.

	LB	UB
v1	0	10
v2	0	10
v3	0	10
v4	0	10
v5	5	5
v6	2.5	5
v7	0	1.25
v8	0	1.25
v9	0	5
v10	0	1.25
v11	0	1.25
v12	0	1.25

The minimum and the maximum range of each reaction flux are shown for the metabolic model in Fig. 1A.

In the second step, given a set of feasible steady-state flux distributions, $F$, we aim to find a non-negative linear combination of sign-fixed variable reactions. According to the Y-model, trade-offs are possible only between fluxes of fixed-sign variable reactions since increasing the flux of one is compensated by a change in fluxes of the other reactions. In contrast, fluxes of sign-variable reactions cannot appear in any trade-offs since their change may not be accounted by compensatory change of flux in the other reactions.

Let the linear combination of fluxes over $F$ be determined by a vector $k$, such that $kN = b$. To enumerate all trade-offs, one can minimize the number of non-zero entries (i.e. the support) of $kN$ that correspond to fixed-sign variable reactions in *F*, denoted by $R_{FS - var,F}$, and at least one fixed reaction in *F* (from the set of reactions denoted by $R_{fixed,F}$). Since minimizing the support is an NP-hard problem^22,23^, we approximated it by minimizing the first norm of $kN$, i.e. $||kN||_{1}$, resulting in the following convex optimization problem:

$$\begin{aligned}& \qquad min\left\| {kN} \right\|_{1} \\ & {\text{s.t.}} \\ &\qquad Nv = 0 \\ &\qquad kN = b \\ & \qquad \sum b_{i} \ge 1, \quad~if \, v_{i} \in R_{{fixed,~F}} \\ &\qquad b_{i} \le 0,if \, v_{i} \in R_{{FS - var,F}} \\ &\qquad b_{i} = 0,if \, v_{i} \notin R_{{FS - var,F}} . \\ \end{aligned}$$

Clearly, then, case in which the applied network constraints do not result in fixed reactions yield no trade-offs, since the Y-model specification cannot be applied. Since we aim to enumerate all trade-offs, the formulation above is modified to exclude all previously found solutions (i.e., trade-offs); this is achieved by using integer cuts^24^. The objective function uses the absolute value function, which can be readily cast as a linear programming (LP) problem^25^, resulting in the implemented mixed-integer LP (MILP) formulation (see Supplementary Note).

To illustrate trade-offs identified by FluTO, we use the metabolic network in Fig. 1A along with its constraints. We identified ten trade-offs, each including only two reactions, as shown in Fig. 1B. For instance, by taking the linear combination of the third, fifth, and eighth row of $N$, i.e. $N_{3 \cdot } + N_{5 \cdot } - N_{8 \cdot }$ we obtain $v_{5} - v_{6} - 2v_{8} = 0$. Therefore, $v_{5} = v_{6} + 2v_{8}$ that corresponds to a two-reaction trade-off since $v_{5}$ is constant in $F$. By inspecting the graph representation of the trade-offs (Fig. 1B), we conclude that reactions $R_{6}$ and $R_{9}$ participate in the largest number of trade-offs, and changes in their steady-state fluxes are propagated in the rest of the network via the trade-off relations.

### Flux trade-offs in *E. coli* using growth and nutrient uptake constraints

**Figure 2:**
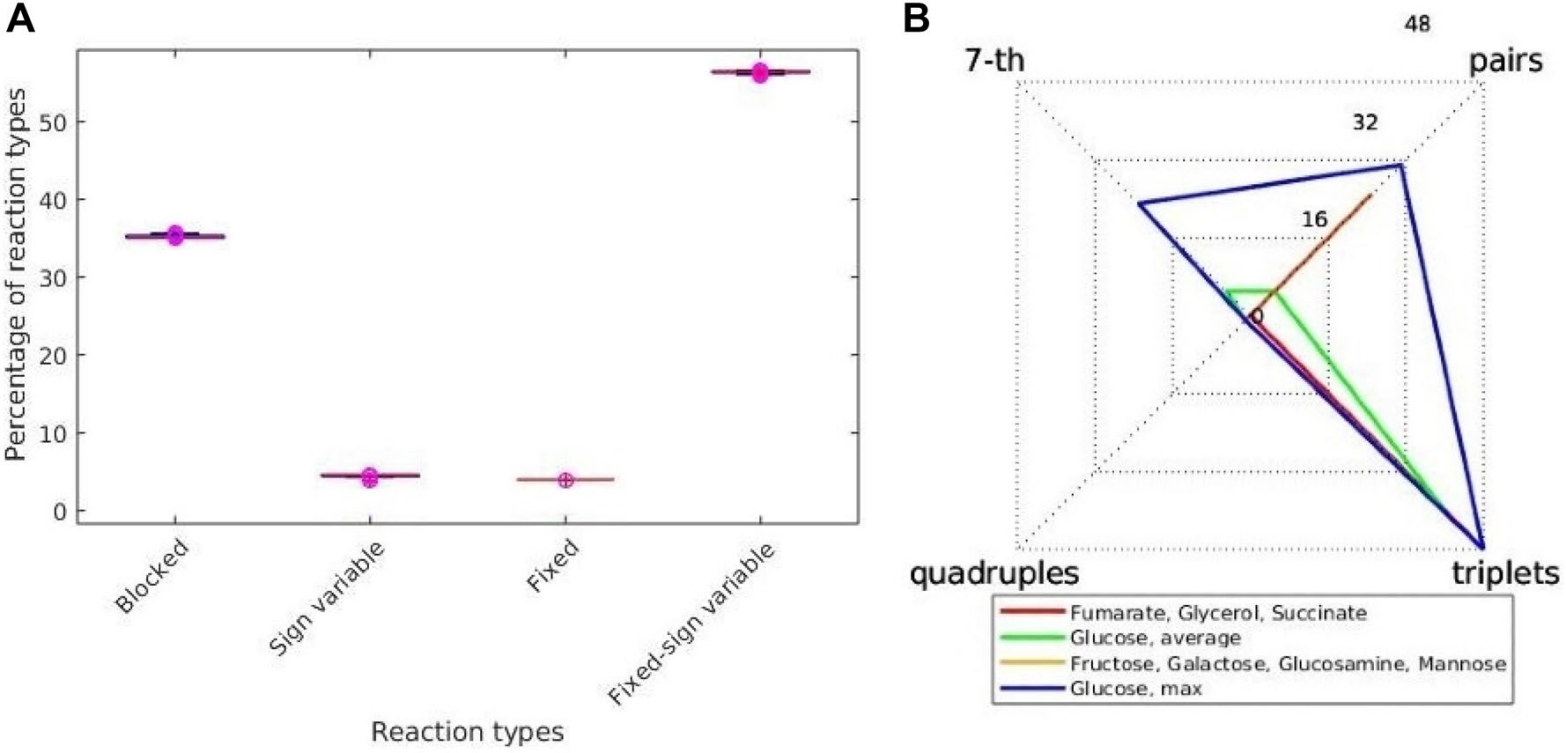
Reaction types based on variability and trade-offs types in *E. coli* under 26 growth conditions. (**A**) Box plots of the four reaction types across the 26 growth conditions. (**B**) Numbers of trade-offs involving different numbers of reactions, two, three, four and seven, for the 26 growth conditions group in four categories, sharing the same numbers.

To determine flux trade-offs in *E. coli,* we applied FluTO with the genome-scale metabolic network model iJO1366^14^ of *E. coli* using data from 29 experiments proving different growth and nutrient uptake constraints^26–28^ . To this end, for each experiment, we first determined the set $F$ of feasible steady-state flux distributions using FVA. We did not identify any fixed reactions with the measured growth rates for three carbon sources, namely acetate, pyruvate, and xylose, precluding us from applying FluTO. For the remaining 26 conditions, we observed that the number of fixed reactions ranged from 101, for three conditions (growth on glucose between 0.11 and 0.2 *h*^−1^), to 103, for the remaining conditions; the number of fixed-sign variable reactions ranged from 1455 for growth rate of 0.11 *h*^−1^ with glucose as a carbon source, to 1462 for growth rate of 0.66 *h*^−1^ with the same carbon source (see Fig. 2A).

Due to the small variability in these numbers over the different carbon sources, we hypothesized that there exist small differences in the determined trade-offs. However, we identified that this was not the case—the smallest number of trade-offs (26) was identified for growth with fructose, galactose, mannose, and glucosamine as carbon sources. At the same time, the largest number of trade-offs (72) was found for a growth rate between 0.21 and 0.31 *h*^−1^ with glucose as a carbon source (Fig. 2B). Thus, we did not identify an association between the growth rate and the number of trade-offs with glucose as a carbon source. However, the growth conditions with the smallest number of trade-offs (i.e., 26 and 32) showed a larger number of pairs of reactions in trade-off than the conditions with the larger total number of trade-offs (i.e., 49 and 72 dominated by trade-offs including three reactions) (see Supplementary Table 6).

We next inspected the reactions that were participating in the identified flux trade-offs. We found that reactions in trade-offs over all considered conditions were involved in cofactor and prosthetic group biosynthesis, namely the ISC and SUF machineries as well as ISC and SUF cysteine desulfuration. The remaining reactions were present in trade-offs in a condition-specific fashion, and included anaplerotic reactions, glycerophospholipid metabolism, glycolysis/gluconeogenesis, and transport/inner membrane metabolic systems (see Supplementary Table 7). These results indicated that FluTO could provide insights into trade-offs that shape the alternative flux routes in the flux space compatible with growth and nutrient uptake constraints.

### Flux trade-offs in *E. coli* and *S. cerevisiae* are specific to carbon sources

The previous analysis aimed to identify trade-offs under fixed uptake and growth rate constraints, which precludes: (i) analysis of the extent to which modeled growth, via the biomass reaction, participates in trade-offs and (ii) tackling the issue of whether trade-offs differ across different carbon sources^14^. To facilitate this analysis, we next determined the flux trade-offs in *E. coli* by using the iJO1366 metabolic model with 61 carbon sources known to support growth in this organism ^18,29^ . To constrain the set of feasible steady-state flux distributions, we fix glucose uptake to 10 mmol.gDW-1 h-1 and determine the maximum flux supported by the ATP synthase phoshphoribosyl transferase, and maintenance reactions using FVA. By fixing the rates of these reactions to the calculated respective maxima, we then determined the maximum rate of phosphate uptake (0.9476 mmol gDW-1 h-1), for which we used a final fixed constraint.

**Figure 3:**
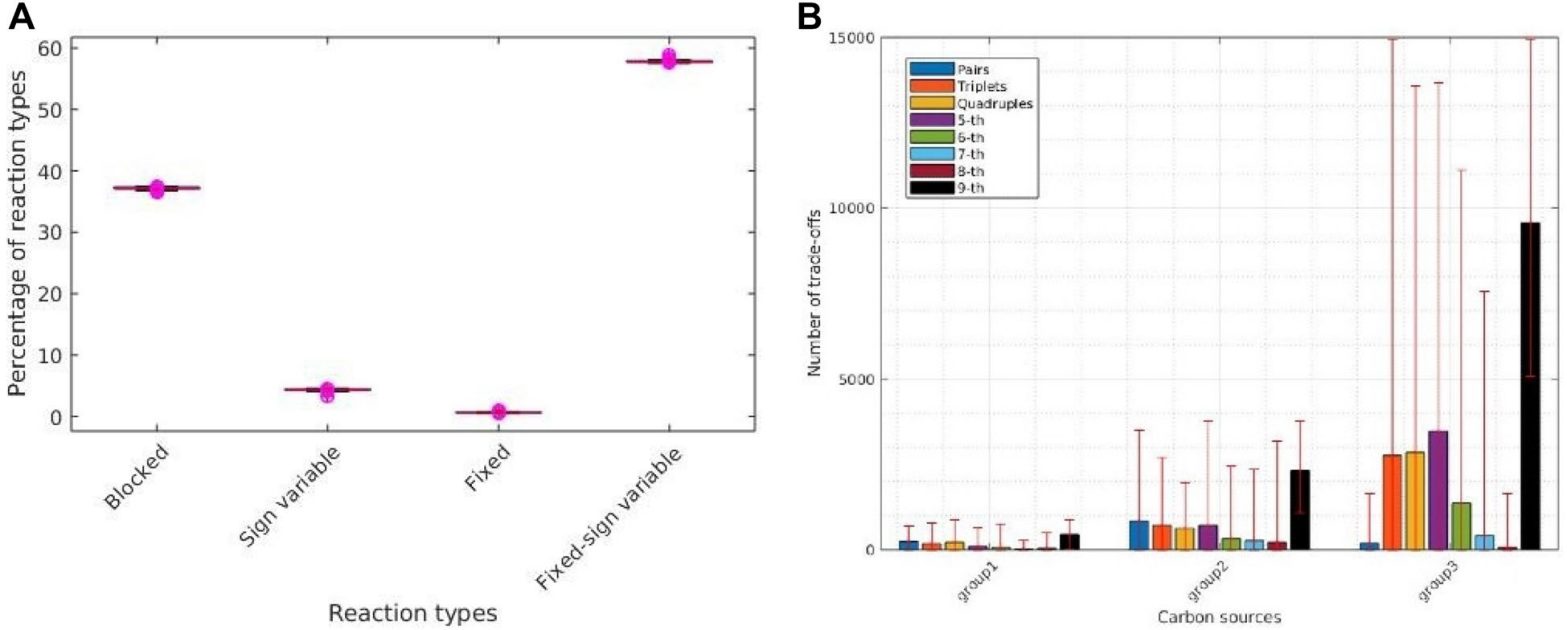
Reaction types based on variability and trade-offs types in *E. coli* grown under different carbon sources. (A) Box plots showing the percentage of the four reaction types across the 53 carbon sources. (B) Numbers of trade-offs involving a different number of reactions for the 53 growth conditions group in three categories, group 1: (S)-Propane-1,2-diol, N-Acetyl-D-mannosamine, Butyrate (n-C4:0), D-Galactose, D-Glucuronate, Maltose, L-Rhamnose, N-Acetylneuraminate, D-Alanine, L-Asparagine, L-Alanine, Octadecenoate (n-C18:1), Melibiose. group 2: Dihydroxyacetone, L-Fucose ,Acetoacetate, Adenosine, 2-Oxoglutarate, Fumarate, Galactitol, L-Glutamine, L-Lyxose, D-Malate, Maltotriose, D-Mannose, Pyruvate, Succinate, Trehalose, L-Aspartate, L-Glutamate, Inosine. group 3: 5-Dehydro-D-gluconate, L-Arabinose, D-Gluconate, L-Lactate, L-Malate, D-Ribose, D-Sorbitol, N-Acetyl-D-glucosamine, D-Allose, Deoxyadenosine, D-Fructose, D-Galactarate, D-Galacturonate, D-Glucose, D-Glucarate, Glycerol, Lactose, Phenylacetaldehyde, L-tartrate, Thymidine, Uridine, D-Xylose.

The imposed constraints did not result in fixed reactions for eight carbon sources, so we determined trade-offs for the remaining 53 different carbon sources (Supplementary Table 8). We observed that the number of fixed reactions ranged from 14 (0.5%), for glucose, to 25 (0.9%), for Phenylacetaldehyd (see Fig. 3A). Unlike the previously analyzed case, here the number of fixed-sign variable reactions differs substantially between carbon sources, with the smallest number of 1488 (57.6%) reactions for Uridine and Inosine, and the largest number of 1520 (58.8%) reactions for Octadecenoate (n-C18:1) as a carbon source (Fig. 3A). Therefore, we hypothesize that the flux trade-offs differ substantially between the investigated carbon sources. We found that growth under D-Galactose, N-Acetyl-D-mannosamine, and D-Glucuronate resulted in the smallest number of flux trade-offs. At the same time, usage D-Galacturonate, D-Galactose, and N-Acetyl-D-glucosamine led to the largest number of flux trade-offs (Fig. 3B). Interestingly, the carbon sources could be divided into three groups associated with a small, moderate, and a large number of flux trade-offs; in addition, we could not identify a particular trend in the contribution of trade-offs of different sizes (determined by the number of participating reactions) (Fig. 3B, Supplementary Figs. 1–3).

Growth, modeled by the flux through the biomass reaction, was found to be in trade-off with a variable number of reactions over the different carbon sources (see Supplementary Table 9). For instance, under glucose (and applied constraints), growth participated in 16 trade-offs, each including three reactions. These reactions belong to the non-mevalonate (MEP) pathway for isoprenoid biosynthesis and also comprise 1-deoxy-D-xylulose 5-phosphate synthase, 1-deoxy-D-xylulose reductoisomerase, Quinolinate synthase, L-Histidine exchange, and transport via diffusion. Interestingly, histidine-requiring *E. coli* strain encountering histidine-rich and starved conditions lost growth fitness under rich conditions^30^. These reactions, along with magnesium, potassium, and calcium exchange, also appeared to be in trade-off with growth for the other carbon sources (see Supplementary Table 10). Altogether, reactions involved in cofactor and cofactor group biosynthesis are involved in trade-offs appearing overall conditions, while those involved in the usage of particular carbon sources are involved in trade-offs under the respective growth scenario (see Supplementary Table 10).

We then applied FluTO to determine flux trade-offs in *S. cerevisiae* by using the yeast genome-scale metabolic model yeastGEMv8.3.3^19^ with 14 carbon sources known to support growth in this organism^18,29^ . To constrain the set of feasible steady-state flux distributions, we fixed the uptake of each carbon source to 10 mmol gDW^−1^ h^−1^ and determined the maximum flux supported by the ATP synthase using FVA. By fixing its rates to the calculated maximum, we then determined the maximum oxygen uptake rate, which we used as a final constraint.

For one carbon source (i.e., L-arabinose), the imposed constraints did not result in fixed reactions, so we determined trade-offs for the remaining 13 carbon sources (Supplementary Table 11 and 12). We observed that the number of fixed reactions ranged from 18 (0.4%), for glucose and fructose, to 26 (0.6%), for xylose (see Supplementary Fig. 4). Unlike the *E. coli* case discussed above, the number of fixed-sign variable reactions was quite similar among carbon sources, with exception of arabinose, ribose, and xylose. However, the flux trade-offs differ substantially between the investigated carbon sources (Supplementary Fig. 5): While the number of flux trade-offs is the smallest for growth under xylose, maltose, and fructose, in line with the smallest number of fixed-sign variable reactions, we found that the carbon sources can be divided into three groups based on the number of flux trade-offs, as in the case of *E. coli* (Supplementary Figs. 1–3). The majority of the reactions participating in trade-offs overall conditions were involved in oxidoreductive processes. Unlike in *E. coli*, growth in *S. cerevisiae* was not involved in any of the determined trade-offs. The reason for this finding is the set of constraints applied to the network, which result in a small range of flux values supported by the biomass reactions, whereby it is excluded from consideration in the flux trade-offs.

### Flux trade-offs in *Arabidopsis thaliana* depend on the availability of key nutrients

**Figure 4:**
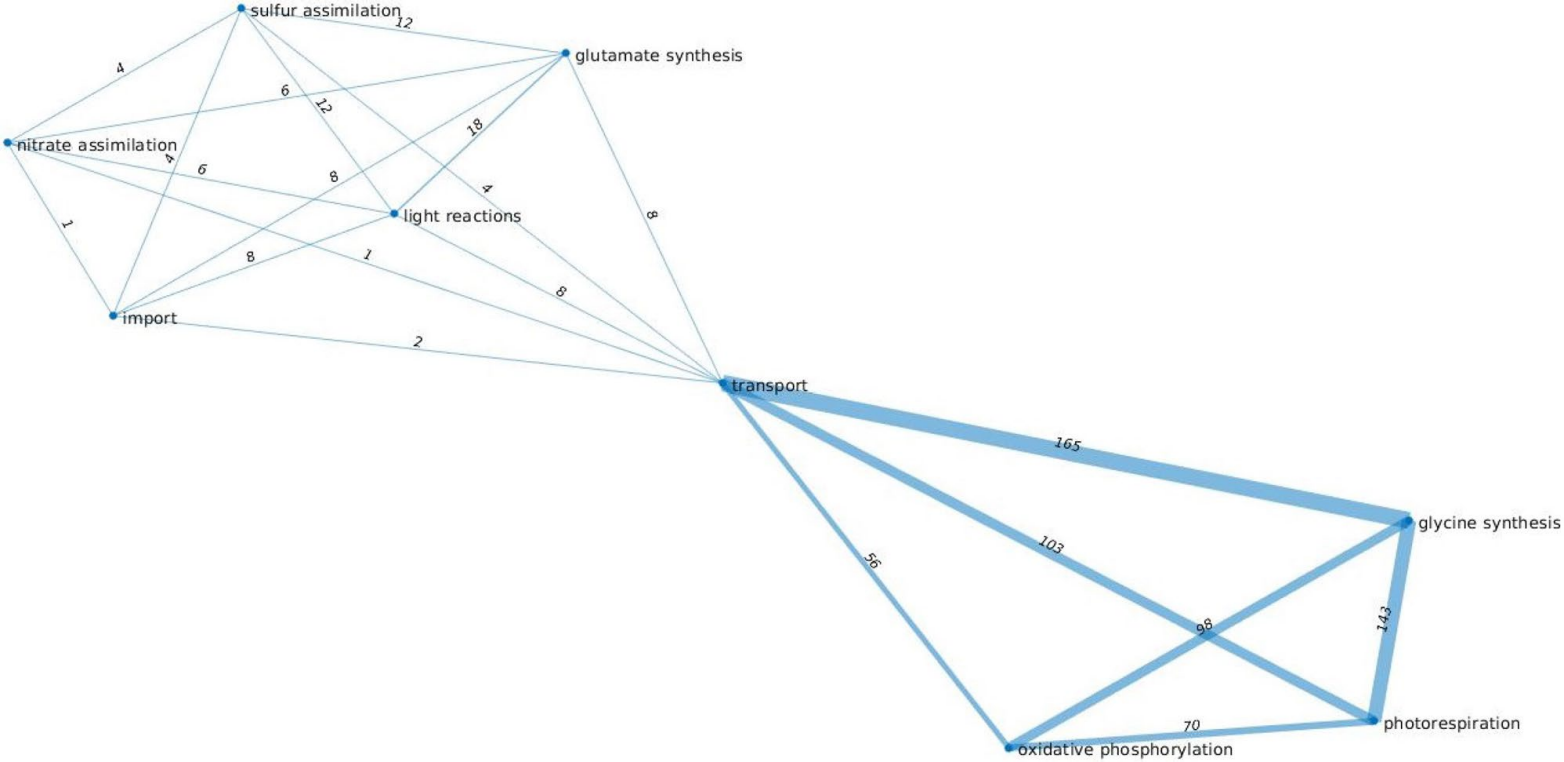
Relation between pathways based on shared trade-offs. A weighted graph shows the number of trade-offs that pathways share with biomass reactions for limiting carbon growth conditions in the model of *A. thaliana*. Here, nodes represent the pathways and the weighted edge between the two nodes shows the number of shared trade-offs between two pathways, determined by the number of reactions from two pathways that participate in the same trade-offs.

We also inspected the extent to which flux trade-offs are affected by using different biomass functions modeling restrictions on the availability of key nutrients. To this end, we employed the AraCore mode of *A. thaliana*, with biomass reactions for optimal nitrogen (light-limiting), limiting nitrogen, and limiting carbon growth conditions^20^. By applying similar constraints for the other approaches (see Methods), we found that the number of flux trade-offs was largely unaffected by the biomass function used (see Supplementary Table 13 and 14). These findings point that the trade-offs are due to metabolites that do not directly participate in the biomass reactions. The majority of the trade-offs can be grouped into two classes; the first involving: light reactions, nitrate assimilation, glutamate synthesis, and sulfur assimilation, and the second comprising: photorespiration, glycine synthesis, oxidative phosphorylation, and transport (see Fig. 4). The former highlight the seemingly tight interrelation between nitrogen and sulfur metabolism in plants^31^, while the latter recap the well-established coupling between processes connecting carbon and nitrogen metabolisms^32^; these results were obtained only by using the structure of the metabolic network along with biochemically meaningful constraints, which further highlights the importance of the identified absolute flux trade-offs.

### Participation of multiple metabolic subsystems in selected trade-offs

To get better understanding of the identified flux trade-offs in the analyzed metabolic networks, we next focused on identifying which trade-offs include reactions that participate in multiple metabolic subsystems, as defined in the models. Trade-offs that include reactions from multiple metabolic subsystems pinpoint mechanisms by which large-scale effects of flux changes can be achieved while respecting network constraints and fulfilling of different tasks.

**Figure 5:**
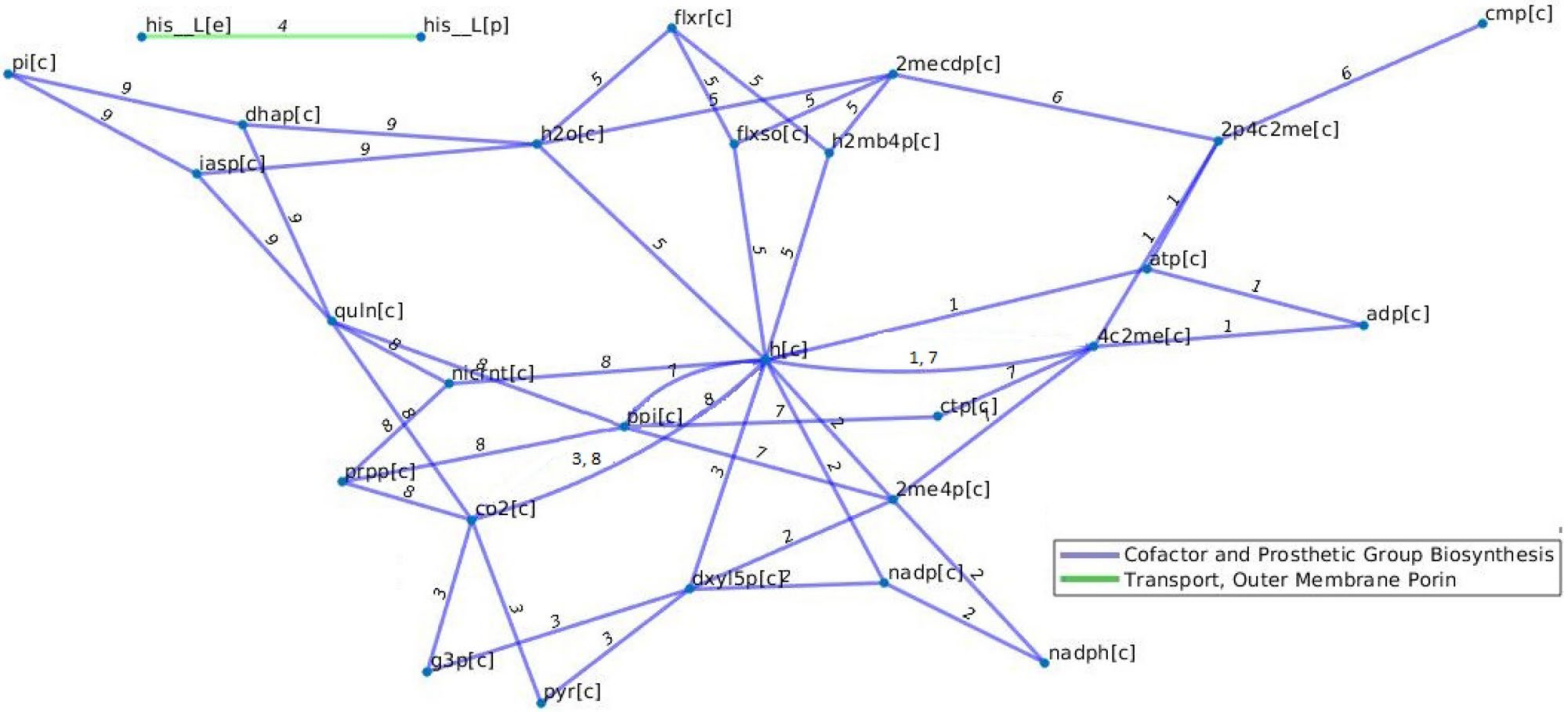
Trade-offs that include the biomass reaction in the model of *E.* coli with glucose as a carbon source. The following reactions are depicted (1) 4-(cytidine 5'-diphospho)-2-C-methyl-D-erythritol kinase, (2) 1-deoxy-D-xylulose reductoisomerase, (3) 1-deoxy-D-xylulose 5-phosphate synthase, (4) L-histidine transport via diffusion (extracellular to periplasm), (5) 2-C-methyl-D-erythritol 4-phosphate cytidylyltransferase, (6) 2C-methyl-D-erythritol 2,4 cyclodiphosphate dehydratase, (7) 2-C-methyl-D-erythritol 2,4-cyclodiphosphate synthase, (8) Nicotinate-nucleotide diphosphorylase (carboxylating), and (9) Quinolinate synthase. Nodes correspond to the following metabolites, adp: adenosine diphosphate, 2me4p: 2-C-methyl-D-erythritol 4-phosphate, 2mecdp: 2-C-methyl-D-erythritol 2,4-cyclodiphosphate, 4c2me: 4-(cytidine 5'-diphospho)-2-C-methyl-D-erythritol, 2p4c2me: 2-phospho-4-(cytidine 5'-diphospho)-2-C-methyl-D-erythritol, ctp: C9H12N3O14P3, atp: adenosine triphosphate, flxr: Flavodoxin reduced, flxso: Flavodoxin semi oxidized, g3p: Glyceraldehyde 3-phosphate, dhap: Dihydroxyacetone phosphate, cmp: cytidine monophosphate, co2: CO2, dxyl5p: 1-deoxy-D-xylulose 5-phosphate, h: H + , h2mb4p: 1-hydroxy-2-methyl-2-(E)-butenyl 4-diphosphate, h2o:water, iasp: Iminoaspartate, nadp: Nicotinamide adenine dinucleotide phosphate, nadph: Nicotinamide adenine dinucleotide phosphate-reduced, nicrnt: Nicotinate D-ribonucleotide, pi: Phosphate, ppi: Diphosphate, prpp: 5-Phospho-alpha-D-ribose 1-diphosphate, pyr: Pyruvate, quln: Quinolinate, his__L: L-Histidine, [c] corresponds to cytoplasm, [p]: periplasm, and [e]: extracellular.

**Figure 6:**
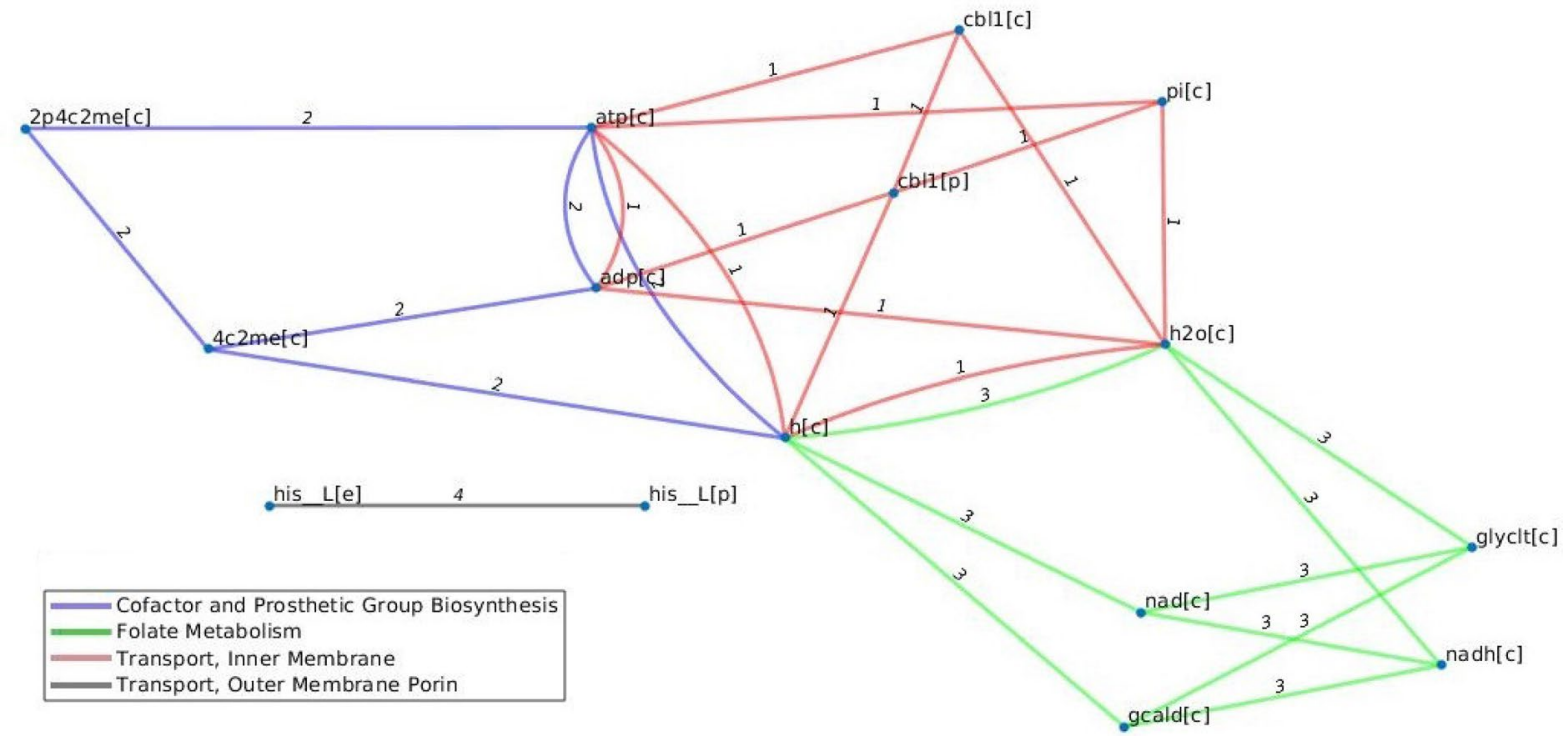
Trade-offs with four reactions in the model of E. coli under different carbon sources. The trade-offs with four reactions with thymidine are also present in the trade-offs with L-Arabinose as a carbon source. The following reactions are included (1) 4-(cytidine 5'-diphospho)-2-C-methyl-D-erythritol kinase, (2) Cob(1)alamin transport via ABC system (periplasm), (3) Glycolaldehyde dehydrogenase, and (4) L-histidine transport via diffusion (extracellular to periplasm). Nodes correspond to the following metabolites, Adp: ADP, 4c2me: 4-(cytidine 5'-diphospho)-2-C-methyl-D-erythritol, 2p4c2me: 2-phospho-4-(cytidine 5'-diphospho)-2-C-methyl-D-erythritol, atp: ATP, cbl1:Cob(I)alamin, gcald: Glycolaldehyde, glyclt: Glycolate, h:H + , h2o: water, nad: Nicotinamide adenine dinucleotide, nadh: Nicotinamide adenine dinucleotide-reduced, pi :Phosphate, his_L: L-Histidine. [c] corresponds to cytoplasm, and [p] corresponds to periplasm.

**Figure 7:**
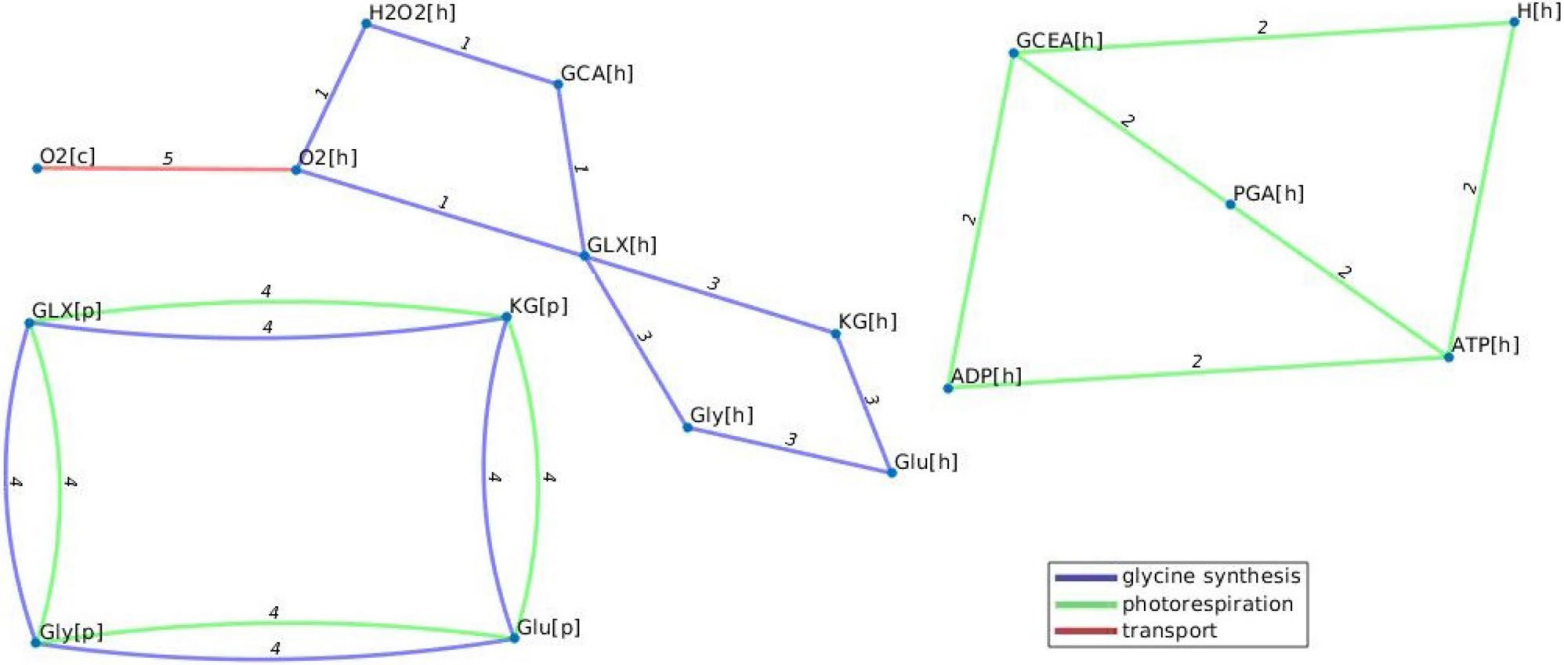
Flux trade-off in the model of *A. thaliana.* The selected trade-off includes reactions from three metabolic subsystems, represented in different colours. The figure shows a trade-off between five reactions. The following reactions are included: (1) Glu:GLX aminotransferase, (2) GCEA kinase, (3) GCA oxidase, (4) Glu:GLX oxidase, aminotransferase and (5) O2 diffusion reaction. The considered metabolites include H: H + /proton, O2: oxygen, ADP: Adenosine diphosphate, ATP: Adenosine triphosphate, PGA: 3-Phosphoglycerate, GCA: Glycolate, GLX: Glyoxylate, Glu: Glutamate, Gly: Glycine, KG: alpha-Ketoglutarate, 2-Oxoglutarate, GCEA: Glycerate, KG: alpha-Ketoglutarate, 2-Oxoglutarate, H2O2: Hydrogen peroxide.[c] corresponds to cytoplasm, [p] :periplasm, and [h]: chloroplast.

To visualize selected trade-offs, we took a metabolite-centric view and showed all metabolites whose corresponding rows of the stoichiometric matrix are used in the derivation of the trade-off; two metabolites are then connected by an edge if they participate in the same reaction. Such a graph representation allowed us to superimpose information about reaction participating in different metabolic subsystems (Figs. 5, 6, and 7). Here we note that each reaction in the metabolic models of *E. coli* participate in only one metabolic subsystem, while this is not the case for the other two models (rendering the analysis more challenging). For this reason, selected trade-offs are visualized only for the models of *E. coli* and *A. thaliana.*

Almost one third of the identified trade-offs in the *E. coli* model include reactions from only one metabolic subsystem. For instance, all trade-offs of size three include reactions from the Cofactor and Prosthetic Group Biosynthesis metabolic subsystem; moreover, all trade-offs with length nine with glutamine as a carbon source include reactions from the Cofactor and Prosthetic Group Biosynthesis and Transport, Outer Membrane Porin metabolic subsystem . Further, all reactions that are in a trade-off with biomass reaction in the *E.coli* model with glucose as a carbon source also belong to Cofactor and Prosthetic Group Biosynthesis and Transport, Outer Membrane Porin pathways (see Fig. 5), indicating the relevance of these pathways in flux re-distribution. Interestingly, we also found that trade-offs of size four in this model are very similar across the different carbon sources (e.g. for thymidine, L-arabinose, and deoxyadenosine, see Fig. 6).

We observed that the maximum number of metabolic subsystems participating in trade-offs was four in the *E. coli* and three for the *A. thaliana* model. For instance, such a trade-off in the model of *A. thaliana* includes photorespiration, glycine synthesis, and transport (Fig. 7), which is present across the three scenarios with different biomass reactions used, documenting the tight relation between carbon and nitrogen metabolism in this model plant.

## Conclusions

Trade-offs are widely present across different levels of biological organization; yet, trade-offs between fluxes have not been studied. Since fluxes are the ultimate phenotype from transcriptional and (post)translational regulation, it is important to understand how trade-offs between them reflect the optimization of one or more cellular objectives. Here, we employ the acquisition-allocation Y-model to develop a two-step constraint-based approach, termed FluTO, that allows enumerating all *absolute* flux trade-offs in a given set of feasible steady-state flux distributions (defined by imposing biochemically meaningful constraints). The idea of FluTO is to determine reactions whose fluxes vary and can be cast as a non-negative linear combination of reactions of fixed flux values in the specific set of flux distributions. In such a way, an increase of one of the fluxes in the non-negative linear combination will impose a decrease in at least one of the others—in line with the concept of trade-offs. This is achieved by using FVA together with a mixed-integer linear program that is used to enumerate all combinations satisfying the imposed criteria.

We used FluTO with constraints on growth and carbon source uptake in *E. coli* to show that flux trade-offs are condition-specific. However, few reactions involved in cofactor and prosthetic group biosynthesis are present in trade-offs across all conditions. To get a better view of growth, modeled by the biomass reaction, is involved in trade-offs with intracellular fluxes in *E. coli,* we used constraints on nutrient uptake and synthesis of ATP. Our results pointed that the flux trade-offs are condition-specific, with growth in trade-offs with the availability of micronutrients, like magnesium, calcium, and potassium. The condition dependence of trade-offs was also found in a similar modeling scenario using *S. cerevisiae*. However, we also found that trade-offs involving the growth, modeled via the biomass reaction, may not be present depending on the constraints applied to the network in the first step of FluTO. Finally, to examine the dependence of trade-offs on the biomass functions used, we investigated three modeling scenarios with a metabolic model of *A. thaliana* to show that the network structure together with the imposed constraints suffices to recover the tight observed couplings between carbon, nitrogen, and sulfur metabolisms.

The proposed formulation of FluTO is rooted in the inter-relation between phenotypic plasticity^33^, robustness^34^, and trade-offs^10^ as seminal network properties. While FluTO currently allows identifying absolute flux trade-offs, the findings depend on the imposed constraints to identify fixed and fixed-sign variable reactions. Although this is biochemically justified by inspecting different growth scenarios, it would also interest to enumerate all *relative* flux trade-offs. These will allow for a more unbiased exploration of trade-offs in a set of feasible steady-state flux distributions. In addition, future work can aim to establish a connection between trade-offs at the level of reaction fluxes and trade-offs between enzyme concentrations by extending the application of FluTO to enzyme-constrained metabolic models^35,36^.

## Supplementary Information

Supplementary Information 1.

Supplementary Information 2.

Supplementary Information 3.

Supplementary Information 4.

Supplementary Information 5.

Supplementary Information 6.

Supplementary Information 7.

Supplementary Information 8.

Supplementary Information 9.

Supplementary Information 10.

Supplementary Information 11.

Supplementary Information 12.

Supplementary Information 13.

Supplementary Information 14.

Supplementary Information 15.
