# Supplementary material for: Identification of flux trade-offs in metabolic networks
Source: Sci Rep. 2021 Dec 10;11:23776. doi: 10.1038/s41598-021-03224-9 (PMC8664830; doi:10.1038/s41598-021-03224-9)
Supplement: Supplementary file 15 — Supplementary Information 15. [file 41598_2021_3224_MOESM15_ESM.docx]

**Supplementary Note.** The MILP formulation to exclude all previously found trade-offs, which is achieved by using integer cuts.

$$min\sum b_{i}{,ifv}_{i}\in R_{FS-var, F}$$

s.t.

$$N_{m\times r}v_{r\times1}=0$$

$$k_{1\times m}N_{m\times r}=b_{1\times r}$$

$${0\leq b}_{i}+Ms_{i}\leq M-1,ifv_{i}\in R_{FS- var. F}$$

$$2\leq\sum s_{i},ifv_{i}\in R_{FS-var.,F}$$

$$1\leq\sum b_{i},ifv_{i}\in R_{fixed, F}$$

$$s_{i}\in\left\{ 0,1 \right\}$$

$$-100\leq b_{i}\leq100,ifv_{i}\in R_{fixed, F}$$

$$-100\leq b_{i}\leq0,ifv_{i}\in R_{FS-var, F}$$

$$b_{i}=0,ifv_{i}\in R_{nonFS-var, F}$$

$$s_{i}\in\left\{ 0,1 \right\},v_{i}\in R_{FS-var, F}$$

$$M=100$$

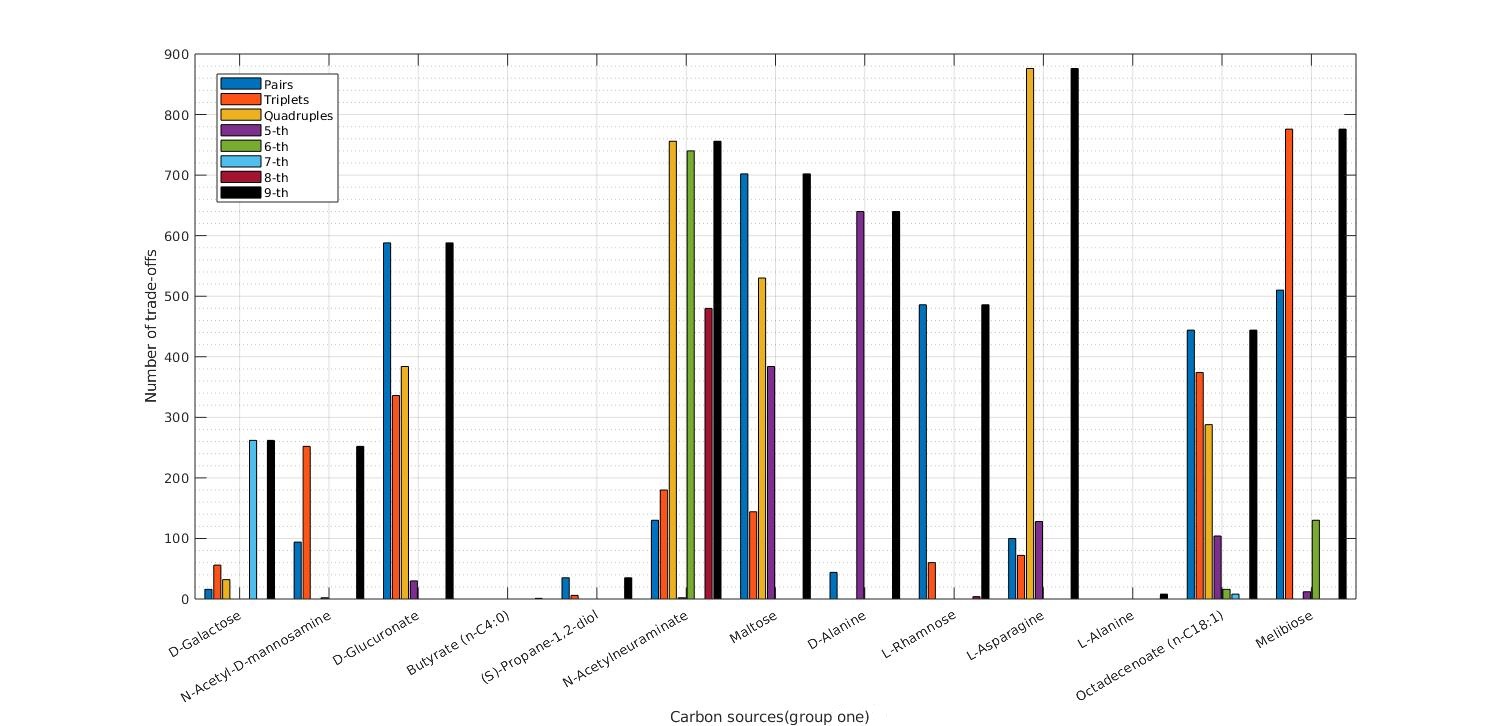


**Figure S1. Number of trade-offs of different sizes in *E. coli* for the first group of carbon sources.** Numbers of trade-offs involving different numbers of reactions, from two to nine, for 19 different carbon sources in the first group for *E. coli*.


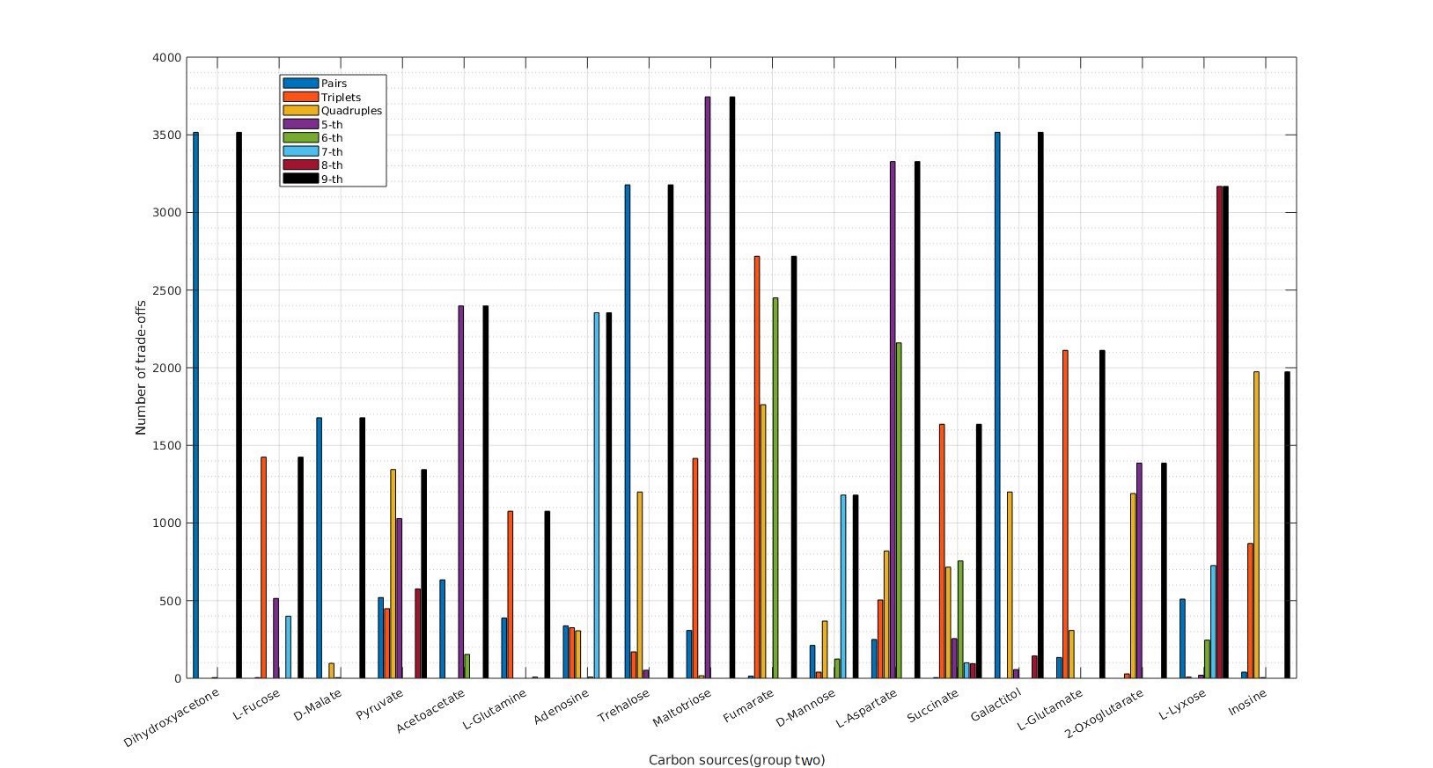


**Figure S2. Number of trade-offs of different sizes in *E. coli* for the second group of carbon sources.** Numbers of trade-offs involving different numbers of reactions, from two to nine, for 18 different carbon sources in the first group for *E. coli*.


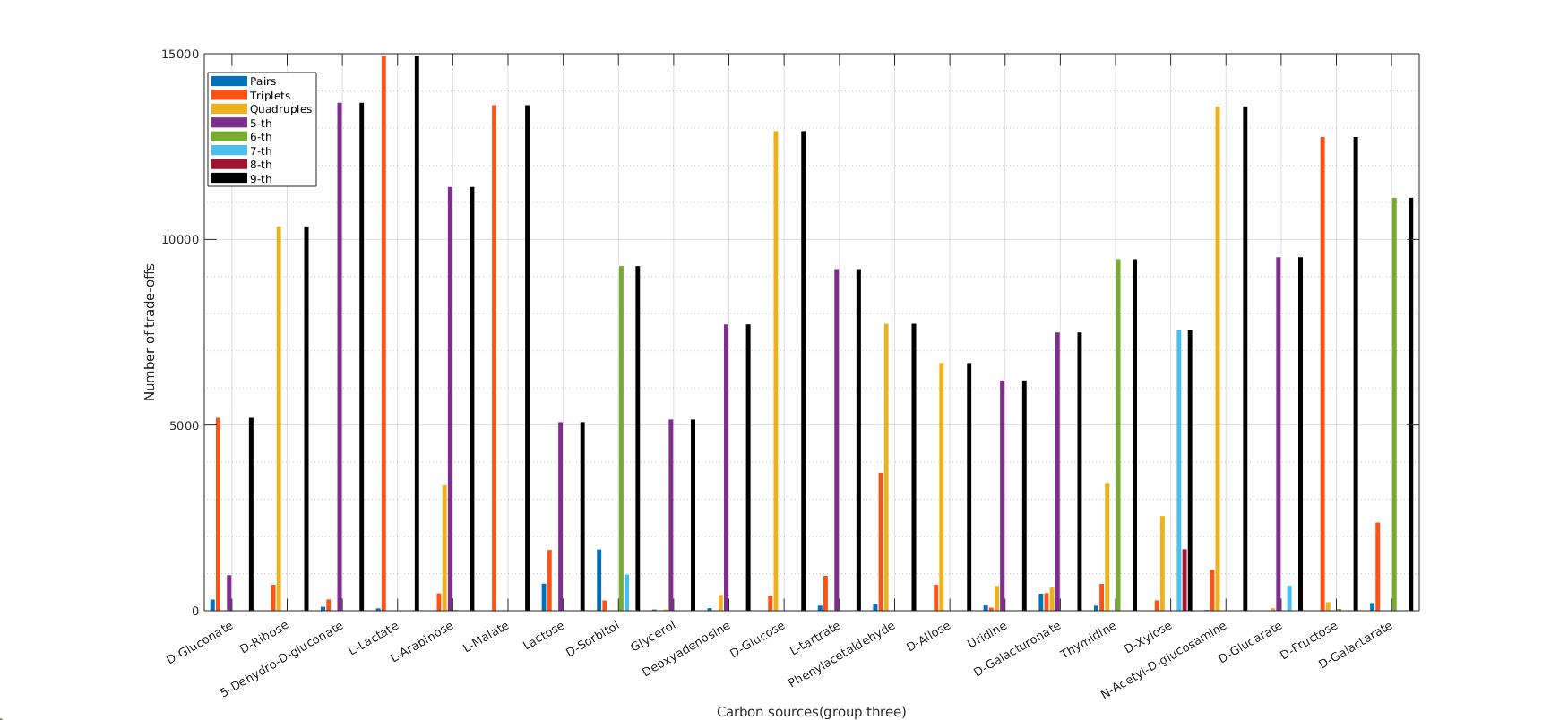


**Figure S3. Number of trade-offs of different sizes in *E. coli* for the third group of carbon sources.** Numbers of trade-offs involving different numbers of reactions, from two to nine, for 15 different carbon sources in the first group for *E. coli*.


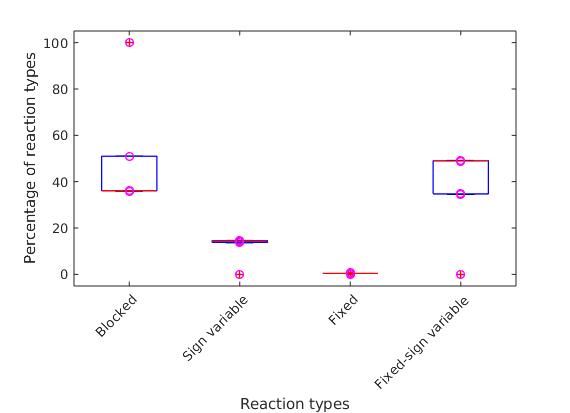


**Figure S4. Reaction types based on variability types in *S. cerevisiae* under 13 different carbon sources.** Box plots of the four reaction types across the 13 carbon sources.

**
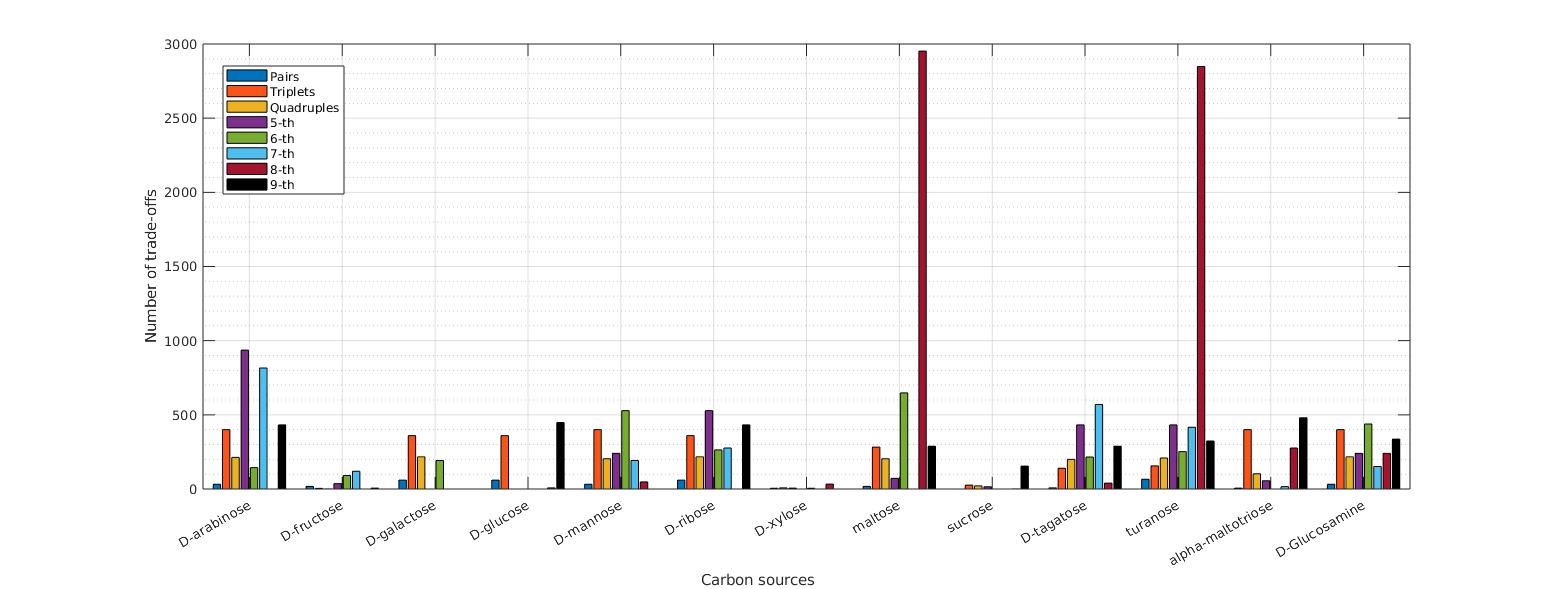
**

**Figure S5. Number of trade-offs of different sizes in *S. cerevisiae* under 13 different carbon sources.** Numbers of trade-offs involving different numbers of reactions, from two to nine, for 13 different carbon sources in *S. cerevisiae*.

**Table S1.** **Reaction types based on FVA in *E. coli* under 26 growth conditions/carbon sources.** The minimum and the maximum range of each reaction flux, with the reaction type are shown for 26 growth conditions/carbon sources in *E. coli*.

**Table S2.** **Reaction types based on FVA in *E. coli* under 53 different carbon sources.** The minimum and the maximum range of each reaction flux, with the reaction type are shown for 53 different carbon sources in *E. coli*.

**Table S3.** **Reaction types based on FVA in *S. cerevisiae* under 13 different carbon sources.** The minimum and the maximum range of each reaction flux, with the reaction type are shown for 13 different carbon sources in *S. cerevisiae*.

**Table S4.** **Reactions grouped for fixed, fixed-sign variable, sign variable, and blocked.** The table shows the reaction type for each model after applying the constraints and FVA.

**Table S5.** **Reaction types based on FVA in *A. thaliana* under three different active biomass reactions.** The minimum and the maximum range of each reaction flux, with the reaction type are shown for three different biomass reactions in *A. thaliana*.

**Table S6.** **Identified trade-offs in the model of *E. coli* under 26 growth conditions/carbon sources.** The table shows the identified trade-offs for 26 growth conditions/carbon sources in *E. coli*. Each column indicates a trade-off.

**Table S7.** **Reactions in the identified trade-offs in the model of *E. coli* under 26 growth conditions/carbon sources.** The table shows the reactions that are participating in the identified trade-offs for 26 growth conditions/carbon sources in *E. coli*. Included are the number of trade-offs in which a reaction participates.

**Table S8.** **Identified trade-offs in the model of *E. coli* for 53 different carbon sources.** The table shows the identified trade-offs for 53 different carbon sources in *E. coli*. Each row indicates a trade-off.

**Table S9.** **Reactions in trade-off with biomass reaction in the model of *E. coli* under 53 different carbon sources.** The table shows the reactions that are participating with biomass reaction in at least one trade-off for 53 different carbon sources in the model of *E. coli*.

**Table S10.** **Reactions in the identified trade-offs for *E. coli* under 53 different carbon sources.** The table shows the reactions that are participating in the identified trade-offs for 53 different carbon sources in *E. coli*. The numbers represent the number of times a reaction participates in identified trade-offs.

**Table S11.** **Identified trade-offs in the model of *S. cerevisiae* under 13 different carbon sources.** The table shows the identified trade-offs for 13 different carbon sources in *S. cerevisiae*. Each row indicates a trade-off.

**Table S12.** **Reactions in the identified trade-offs in the model of *S. cerevisiae* under 13 different carbon sources.** The table shows the reactions that are participating in the identified trade-offs for 13 different carbon sources in *S. cerevisiae*. Included are the number of trade-offs in which a reaction participates.

**Table S13.** **Identified trade-offs in the model of *A. thaliana* under three different active biomass reaction.** The table shows the identified trade-offs under three different active biomass reactions. Each column indicates a trade-off.

**Table S14.** **Reactions in the identified trade-offs in the model of *A. thaliana* under three different active biomass reactions.** The table shows the reactions that are participating in the identified trade-offs for three different biomass reactions in *A. thaliana*. Included are the number of trade-offs in which a reaction participates.
